# Use of haplotypes to identify regions harbouring lethal recessive variants in pigs

**DOI:** 10.1186/s12711-017-0332-3

**Published:** 2017-07-14

**Authors:** David M. Howard, Ricardo Pong-Wong, Pieter W. Knap, John A. Woolliams

**Affiliations:** 1 The Roslin Institute, R(D)SVS, The University of Edinburgh, Midlothian, UK; 2 Genus-PIC, Ratsteich 31, 24837 Schleswig, Germany; 3 Genus PLC, 100 Bluegrass Commons Blvd, Suite 2200, Hendersonville, TN 37075 USA

## Abstract

**Background:**

Lethal recessive genetic variants are maintained at relatively low frequencies in a population in the heterozygous state, but by definition are fatal and therefore unobserved in the homozygous state. Since haplotypes allow the tagging of rare and untyped genetic variants, they have potential for studying lethal recessive variants. In this study, we used a large commercial population to identify putative lethal recessive haplotypes that impact either the total number born (TNB) or the number born alive (NBA) as a proportion of the total number born (NBA/TNB). We also compared the use of haplotypes with a single nucleotide polymorphism (SNP)-by-SNP approach and examined the benefits of using additional haplotypes imputed from low-density genotype data for the detection of lethal recessive variants. Candidate haplotypes were identified using population-wide haplotype frequencies and within-family analyses. These candidate haplotypes were subsequently assessed for putative lethal recessive effects on TNB and NBA/TNB by comparing carrier-to-carrier matings with carrier-to-non-carrier matings.

**Results:**

Using both medium-density and imputed low-density genotype data six regions were identified as containing putative lethal recessive haplotypes that had an effect on TNB. It is likely that these regions were related to at least four putative lethal recessive variants, each located on a different chromosome. Evidence for putative lethal recessive effects on TNB was found on chromosomes 1, 6, 10 and 14 using haplotypes. Using haplotypes from individuals genotyped only at medium-density or a SNP-by-SNP approach did not detect any lethal recessive effects. No lethal recessive haplotypes or SNPs were detected that had an effect on NBA/TNB.

**Conclusions:**

We show that the use of haplotypes from combining medium-density and imputed low-density genotype data is superior for the identification of lethal recessive variants compared to both a SNP-by-SNP approach and to the use of only medium-density data. We developed a formal statistical framework that provided sufficient power to detect lethal recessive variants in species, which produce large full-sib families, while reducing false positive or type I errors. Applying this framework results in improvements in reproductive performance by purging lethal recessive alleles from a population in a timely and cost-effective manner.

**Electronic supplementary material:**

The online version of this article (doi:10.1186/s12711-017-0332-3) contains supplementary material, which is available to authorized users.

## Background

Prenatal mortality impacts the efficacy and profitability of livestock breeding programs. In pigs, embryonic losses are estimated at 30% [1], with a further 10% loss due to foetal mortality [2]. Most (if not all) studies on prenatal mortality in pigs have examined these losses in the context of the dam’s genotype [3–5]. In addition to prenatal losses that are attributable to genetic effects, losses can also occur as a result of environmental factors, including infectious disease, nutrition, hormonal or biochemical effects [6–10]. While deleterious nutritional, hormonal and biochemical effects may be caused or influenced by the sow’s genotype, from the perspective of the developing embryo, they are environmental effects. In livestock populations with small to moderate population sizes, lethal recessive variants have the potential to drift more rapidly to higher frequencies, which increases the risk of losing otherwise viable offspring, with an impact on overall reproductive success.

Historically, pedigree analysis was used to provide evidence of recessive effects [11]. Following the introduction of routine genotyping, genetic markers offered the possibility of identifying lethal recessive variants that underlie observed perinatal mortality [12, 13]. However, for the successful use of molecular genetic marker information to detect a lethal recessive variant causing prenatal mortality, a sufficient level of linkage disequilibrium between the marker allele(s) and the causal variant(s) is required. An alternative approach is to use haplotypes to improve the tagging of rare and untyped variants that are not included on the single nucleotide polymorphism (SNP) genotyping array that is used. The phasing of genotype data provides greater clarity of haplotype heterogeneity and allows the inference of the haplotypes of non-genotyped ancestors and of animals that have been genotyped using lower density arrays. Haplotypes have been successfully used in cattle to identify associations with prenatal mortality [14–16] and a lethal recessive haplotype that is nominally associated with the number of stillborn piglets has also been identified in a study with a smaller population than the one used in our study [17].

The objective of our study was to develop a formal test for detecting haplotypes that are associated with a lethal recessive effect within a pig population. Based on data on haplotypes that were never observed in the homozygous state, candidate haplotypes were identified, using either their haplotype frequency in the population or the lack of homozygous offspring from matings that involve carriers. Then, we examined the top candidate haplotypes for their effect on the total number born (TNB) and the number born alive expressed as a proportion of TNB (NBA/TNB) to identify putative lethal recessive haplotypes. This methodology offers a robust approach for the detection of lethal recessive haplotypes that cause prenatal mortality. More importantly, the ability to detect these lethal recessive haplotypes facilitates selection to remove such haplotypes and reduce prenatal losses within the pig breeding industry. We compared the efficacy of using haplotypes of multiple lengths to detect lethal recessive variants to that of a SNP-by-SNP approach and investigated the benefits of including imputed data from individuals genotyped on a low-density chip.

## Methods

### Study population

For this study, we used a long established closed nucleus line from a commercial pig breeding operation. Pedigree information was available for 3,089,515 individuals born between 3 January 1995 and 10 December 2013. This line consisted of purebred Large White pigs that are principally selected for maternal traits. The mean of TNB was 11.41 (SD = 3.04) and the mean of NBA/TNB was 0.93 (SD = 0.12).

A total of 5660 individuals (3404 males and 2256 females) were genotyped at medium-density (MedD) using the PorcineSNP60 BeadChip (Illumina, San Diego, CA, USA) and a further 12,534 animals (10,638 males and 1896 females) were genotyped using a low-density (LowD) chip containing 402 SNPs. The earliest individual to be genotyped was born on 18 June 1998. The draft pig genome sequence, Sscrofa10.2 [18], was used for ordering and positioning SNPs.

Prior to phasing and imputation, autosomal SNPs with a call rate lower than 90% within the MedD individuals were excluded, leaving 52,238 SNPs. Phasing and imputation of haplotypes was conducted using AlphaImpute v1.21 [19]. MedD individuals with a SNP call rate per animal higher than 95% for a particular chromosome were assigned to a phasing group to which long range phasing [20] was applied. For this group, additional quality control was performed to improve phasing accuracy by removing SNPs with a call rate lower than 97.5%. The number of individuals assigned to the phasing group depended on the call rate specific to that chromosome and varied from 5197 to 5267 individuals. The phases for LowD individuals and for non-genotyped direct ancestors of genotyped individuals were then imputed, which resulted in 23,327 individuals (14,882 males and 8445 females) that were phased and imputed for 47,704 SNPs.

Window sizes of 3, 5 and 10 SNPs were used to determine the phased variants for each haplotype. We applied a sliding window approach by moving one SNP at a time along each autosomal chromosome. Since only haplotypes that had a lethal recessive effect were of interest, haplotypes that were observed in the homozygous form were excluded.

### Statistical approach

We used a two-step process to identify putative lethal recessive haplotypes. Step 1 identified candidate haplotypes and Step 2 tested these candidate haplotypes for a putative lethal recessive effect on the phenotype.

#### Step 1

We used three methods (1A, 1B and 1C) within Step 1 to identify candidate haplotypes. Step 1A used population-wide haplotype frequencies and Steps 1B and 1C used within-family information.

*Step 1A Using population*-*wide haplotype frequency to identify candidate haplotypes.* The expected number of homozygotes ($E[ H ]$) was calculated using the frequency of the haplotype in the population ($q$) as:

1 $$E[ H ] = q^{2} N,$$

where *N* is the total number of haplotyped individuals in the population. The Poisson distribution was used to calculate the probability of observing no homozygous haplotypes $\left( {O[ H ]} \right)$ given $E[ H ]$:

2 $$P (O[ H ] = 0 | E[ H ]) = e^{ - E[ H ]} .$$

Those haplotypes with $P (O[ H ] = 0 |E[ H ])$ < 0.05 were classified as candidate haplotypes. This value was applied since it is the conventional threshold for nominal statistical significance.

*Step 1B Using within*-*family information from carrier sires and carrier dams to identify candidate haplotypes.* Mendelian segregation provides an expectation for the inheritance of alleles from parent to offspring. For matings between any two carriers of a haplotype that failed to produce any offspring that were homozygous for that haplotype, a test against the number of homozygous offspring expected was made. First, we assessed this by using all matings between carrier sires (*S*) and carrier dams (*D*); for $n$ haplotyped offspring produced, the probability of not observing any homozygous offspring was:

3 $$P\left( {O[ H ] = 0 | S, D\;Carriers} \right) = 0.75^{n} .$$

Those haplotypes with $P\left( {O[ H ] = 0 | S, D\;Carriers} \right)$ < 0.05 were classified as candidate haplotypes.

*Step 1C Using within*-*family information from carrier sires and carrier maternal grand sires to identify candidate haplotypes.* To include matings to dams that were not genotyped, the within-family approach was also applied to haplotyped offspring that were produced from carrier sires and from dams sired by carrier maternal grand sires (*MGS*), which adds two levels of complexity i.e. the uncertainty about the haplotypes carried by the maternal grand dam (*MGD*) and uncertainty about the haplotypes inherited by the dam. To predict the likelihood of the *MGD* being a carrier, as above we used the frequency of the haplotype in the population ($q$), with the haplotypes of the dam being predicted based on Mendelian inheritance. The probability of not observing a homozygous offspring within each of the litters resulting from carrier sires and carrier MGS, assuming a null hypothesis of non-lethality was calculated as:

4 $$P\left( {O[ H ] = 0 | S, MGS\;Carriers} \right) = \mathop \prod \limits_{m = 1}^{M} \left( {\frac{{0.5^{{n_{m} }} q + 0.75^{{n_{m} }} + \left( {1 - q} \right) }}{2}} \right),$$

where $M$ is the total number of litters and litter $m$ has $n_{m}$ haplotyped offspring. The derivation of Eq. (4) is in Additional file 1. Haplotypes with $P\left( {O[ H ] = 0 | S, MGS\,Carriers} \right)$ < 0.05 were classified as candidate haplotypes.

#### Step 2 Phenotypic criteria to identify putative lethal recessive haplotypes

The candidate haplotypes that were identified within Step 1 and that were within 3 Mb of one another, were grouped into regions, resulting in 154 regions. The boundaries of each region were defined by the positions of the outermost candidate haplotypes. The haplotype within each region with the lowest probability $P\left( {O[ H ]} \right) = 0$ across Steps 1A, 1B and 1C was then assessed for a lethal recessive phenotypic effect on TNB and NBA/TNB by comparing the outcomes of carrier-to-carrier matings (*C* × *C*), in which prenatal mortality might be anticipated, with carrier-to-non-carriers matings (*C* × *NC*) as a control. The non-carrier could be either the sire or the dam. The comparison between the outcomes of *C* × *C* and *C* × *NC* matings is hereafter referred to as mating status (MS).

For each assessed candidate haplotype, we constructed a linear mixed model to assess the effect and statistical significance of MS upon each trait:

5 $${\mathbf{y}} = {\mathbf{MS}} + {\mathbf{X}}{\varvec{\upbeta}} + {\mathbf{Z}}_{1} {\mathbf{u}} + {\mathbf{Z}}_{2} {\mathbf{v}} + {\mathbf{Z}}_{3} {\mathbf{w}} + {\varvec{\upvarepsilon}} ,$$

where ${\mathbf{y}}$ is the vector of observations for the trait; ${\varvec{\upbeta}}$ is the vector of fixed effects including dam parity number and birthdate of the litter; ${\mathbf{u}}$ is the vector of the genetic effects of the dam, which is assumed to be random and distributed as MVN(0, ${\mathbf{A}}{\varvec{\upsigma}}_{\varvec{u}}^{2}$), where ${\mathbf{A}}$ is the numerator relationship matrix; ${\mathbf{v}}$ is the vector of the effects of the service sire, which is assumed to be random and distributed as MVN(0, **I** ${\varvec{\upsigma}}_{\varvec{v}}^{2}$); ${\mathbf{w}}$ is the vector of the non-genetic effects of the dam, which is assumed to be random and distributed as MVN(0, **I** ${\varvec{\upsigma}}_{\varvec{w}}^{2}$); and ${\varvec{\upvarepsilon}}$ is the vector of random residual effects, which is assumed to be distributed as MVN(0, **I** ${\varvec{\upsigma}}_{{\varvec{\upvarepsilon}}}^{2}$). ${\mathbf{X}}$, ${\mathbf{Z}}_{1}$, ${\mathbf{Z}}_{2}$ and ${\mathbf{Z}}_{3}$ are incidence matrices for the associated effects. A spline [21] was fitted for the birthdate of the litter to allow flexible modelling of the time trend. Since each comparison of MS was done by using only a subset of the data, the variance components were estimated using the whole dataset without fitting MS (see Table 1).

**Table 1:** Phenotypic variances and proportions of phenotypic variance explained by dam, service sire, and maternal effects for total number born (TNB) and number born alive as a proportion of TNB (NBA/TNB), with standard errors in parentheses

Parameter		TNB	NBA/TNB
Phenotypic variance	${{\upsigma }}_{p}^{2}$	9.218	0.014
Dam (genetic)	${{\upsigma }}_{u}^{2}$/${{\upsigma }}_{p}^{2}$	0.097 (0.003)	0.115 (0.008)
Service sire	${{\upsigma }}_{v}^{2}$/${{\upsigma }}_{p}^{2}$	0.053 (0.053)	0.023 (0.002)
Dam (non-genetic)	${{\upsigma }}_{w}^{2}$/${{\upsigma }}_{p}^{2}$	0.071 (0.003)	0.000 (0.000)

The phenotypic variance was estimated as ${{\upsigma }}_{p}^{2} = {{\upsigma }}_{u}^{2} + {{\upsigma }}_{v}^{2} + {{\upsigma }}_{w}^{2} + {{\upsigma }}_{{{\upvarepsilon }}}^{2}$

Statistical significance of MS was judged based on the Wald F-statistics generated by using ASReml 3.0 [22]. To account for the multiple-testing of the 154 haplotypes identified in Step 1, a *P* value below 3.25 × 10^−4^ was required to reach Bonferroni-corrected significance. The nominal significance of MS is reported in the Results section and it is highlighted where it survives multiple-testing.

Following the phenotypic analysis of the sire and dam (*S* × *D*), Model (5) was repeated with the sire and MGS (*S* × *MGS*) classified as carriers or non-carriers, using dam as a factor in the model.

Two criteria had to be met to classify a haplotype as having a putative lethal recessive effect. First, MS had to be statistically significant at the nominal 5% level; and second, the relative reduction ($R$) of litter size between *C* × *NC* and *C* × *C* matings, which was calculated as:

6 $$R = \frac{A}{B},$$

where $A$ is the difference between the predicted mean litter sizes of *C* × *C* and *C* × *NC* and $B$ is the predicted mean litter sizes of *C* × *NC*, had to be within 1.96 standard errors of the expectation for a lethal recessive effect, which was 0.25 for *S* × *D*, and 0.125 for *S* × *MGS*. An approximation of the standard error (SE) of $R$ was calculated [23] as:

7 $$s.e. = \sqrt {\left[ { \frac{A}{B} } \right]^{2} \left[ { \frac{{\sigma_{A}^{2} }}{{A^{2} }} + \frac{{\sigma_{B}^{2} }}{{B^{2} }} - \frac{{2cov\left( {A, B} \right)}}{AB} } \right]} ,$$

where $\sigma_{A}^{2}$ and $\sigma_{B}^{2}$ denote the predicted variances of $A$ and $B$, respectively. The appropriate means and sampling co(variances) were obtained from fitting Model (5) using ASReml 3.0 [22].

### Multiple putative lethal recessive haplotypes on a single chromosome

In the event that two distinct regions containing putative lethal recessive haplotypes were on the same chromosome, then the effect of being a carrier for both haplotypes was assessed using the phenotypic analyses described previously in Step 2. However, in this case, for the categorisation of MS, *C* × *C* indicates a double carrier individual mated to another double carrier and *C* × *NC* indicates a double carrier mated to an individual that carried neither haplotype. As previously, phenotypic assessment was carried out using both *S* × *D* and *S* × *MGS* models. Pairwise linkage disequilibrium (*r* ^2^) between the two haplotypes and the SNPs located within the haplotypes was calculated by using only the 5660 individuals genotyped on the MedD panel.

### Comparison with a SNP-by-SNP approach

To evaluate the effectiveness of the haplotype approach described above, the same Steps and methods were repeated for single SNPs using all 23,327 individuals, including those for which genotypes were imputed. SNPs that were never observed in the homozygous state for the minor allele were assessed using the three methods in Step 1 to identify candidate SNPs. Then, for these candidate SNPs, *C* × *C* matings were compared to *C* × *NC* matings and a reduction in TNB or NBA/TNB was tested by using the phenotypic analyses of Step 2.

### Comparison with only MedD individuals

An assessment of the comparative effectiveness of using only individuals genotyped on the MedD chip was also conducted, by restricting the analysis to the 5660 individuals that were genotyped on that chip and their phased haplotypes that were generated by AlphaImpute v1.21 [19]. The two Steps and methods described above were repeated with 3, 5 and 10-SNP haplotypes, and also SNP-by-SNP.

## Results

### Identification of candidate haplotypes

**Fig. 1:**
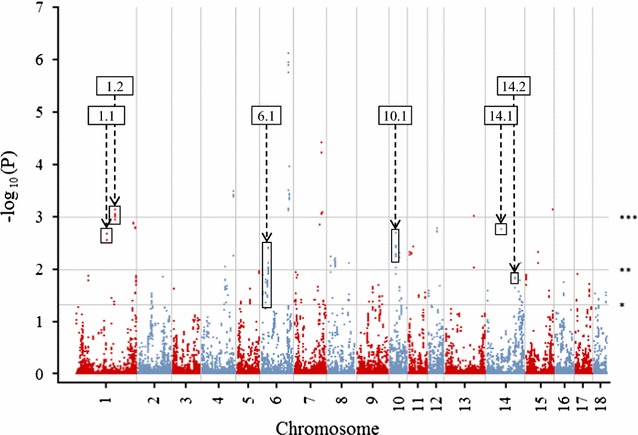
Probability of observing no homozygous offspring given the expected number of homozygous individuals within the population ($P (O[H] = 0 |E[H])$) using 5-SNP long haplotypes. Regions identified as containing putative lethal recessive haplotypes are indicated. The positions of SNPs on each chromosome are in Mb. *P* values are nominal, *P < 0.05; **P < 0.01; ***P < 0.001

Step 1A identified 1426 candidate haplotypes with $P (O[ H ] = 0 |E[ H ])$ less than 0.05. A Manhattan plot of −log_10_  $P (O[ H ] = 0 |E[ H ])$ for the 5-SNP haplotypes against chromosomal position is in Fig. 1 and the regions that were ultimately identified as containing putative lethal recessive haplotypes are highlighted. Step 1B identified 2072 candidate haplotypes from matings of carrier sires and carrier dams with $P\left( {O[ H ] = 0 | S, D\;Carriers} \right)$ less than 0.05, and Step 1C identified 902 candidate haplotypes from matings defined by carrier sires and carrier MGS with $P\left( {O[ H ] = 0 | S, MGS\;Carriers} \right)$ less than 0.05. The within-family analysis of the haplotyped offspring from carrier sires and carrier MGS (Step 1C) provided a larger number of offspring than the analysis of haplotyped offspring from carrier sires to carrier dams (Step 1B). However, the probability that the offspring considered in Step 1C were homozygous was lower compared to that in Step 1B. There were 154 regions that contained haplotypes that were significant at the 5% level in Step 1 with the most significant haplotype within each region assessed for a lethal recessive effect on TNB and on NBA/TNB.

### Putative lethal recessive haplotypes with an effect on TNB

For a haplotype to be assigned a putative lethal recessive status, MS had to be nominally statistically significant within Model (5) and $R$ had to be within 1.96 standard errors of the expected lethal effect. Tables 2 and 3 summarise the statistical significance of the putative lethal recessive haplotypes with an effect on TNB using the *S* × *D* model and the *S* × *MGS* model, respectively. Locations of the regions and locations and frequencies of the putative lethal recessive haplotypes identified are in Table 4.

**Table 2:** Regions containing putative lethal recessive haplotypes with an effect on total number born using the *S* × *D* model

Region	Step 1A	Step 1B	Step 2, *S* × *D* model			
	$P (O[ H ] = 0 | E[ H ])$	$P (O[ H ] = 0 | S,D \;Carriers)$	*P* value of MS	$R$	Number of SE from the expectation of a lethal recessive effect	Number of SE from no effect
6.1	7.84 × 10^−4^	1.00 × 10^−2^	5.62 × 10^−2^	0.157	1.13	1.91
10.1	5.30 × 10^−3^	1.78 × 10^−3^	4.79 × 10^−2^	0.157	1.18	1.97
*14.1*	*1.77* × *10* ^−*3*^	*1.34* × *10* ^−*5*^	*2.03* × *10* ^−*5*^	*0.190*	*1.34*	*4.24*
14.2	1.50 × 10^−2^	5.64 × 10^−3^	9.71 × 10^−4^	0.196	0.91	3.31
*14.1 and 14.2* ^*†*^			*1.02* × *10* ^−*4*^	*0.240*	*0.16*	*3.93*

Regions containing a putative lethal recessive haplotype are named based on the chromosome and their order along that chromosome, i.e. 6.1 refers to the first region containing a putative lethal recessive haplotype located along chromosome 6. *P* values are given for the probability of observing no homozygotes within the population given the number expected based on the frequency of the haplotype ($P (O[ H ] = 0 |E[ H ])$), the probability of observing no homozygous offspring given the expected number from matings between a carrier sire and a carrier dam ($P\left( {O[ H ] = 0 | S, D\;Carriers} \right)$) and the significance of mating status (MS) using the *S* × *D* model. $R$ is the relative reduction in total number born as a result of matings between two carriers compared to matings between a carrier and a non-carrier. The number of standard errors that $R$ was from the expectation of a lethal recessive effect and from no effect are given. *P* values are nominal, with the haplotypes that achieved Bonferroni corrected significance (*P* < 3.25 × 10^−4^) for MS highlighted in italics. Results arising from the concurrent assessment of two putative lethal recessive haplotypes are denoted by ^†^

**Table 3:** Regions containing putative lethal recessive haplotypes with an effect on total number born using the *S* × *MGS* model

Region	Step 1A	Step 1C	Step 2, *S* × *MGS* model			
	$P (O[ H ] = 0 | E[ H ])$	$P (O[ H ] = 0 | S,MGS\;Carriers)$	*P* value of MS	$R$	Number of SE from the expectation of a lethal recessive effect	Number of SE from no effect
*1.1*	*2.15* × *10* ^−*3*^	*3.37* × *10* ^−*1*^	*6.99* × *10* ^−*5*^	*0.092*	*1.39*	*3.95*
1.2	1.17 × 10^−3^	2.89 × 10^−1^	7.11 × 10^−3^	0.070	2.13^‡^	2.68
*1.1 and 1.2* ^*†*^			*2.07* × *10* ^−*4*^	*0.122*	*0.09*	*3.68*
6.1	7.84 × 10^−4^	4.61 × 10^−1^	3.25 × 10^−3^	0.103	0.62	2.93
10.1	5.30 × 10^−3^	4.74 × 10^−6^	7.69 × 10^−3^	0.083	1.37	2.65
*14.1*	*1.77* × *10* ^−*3*^	*8.67* × *10* ^−*3*^	*6.28* × *10* ^−*10*^	*0.101*	*1.45*	*6.04*
*14.2*	*1.50* × *10* ^−*2*^	*1.93* × *10* ^−*2*^	*1.01* × *10* ^−*5*^	*0.084*	*2.11* ^*‡*^	*4.36*
*14.1 and 14.2* ^*†*^			*2.97* × *10* ^−*6*^	*0.097*	*1.33*	*4.59*

Regions containing a putative lethal recessive haplotype are named based on the chromosome and their order along that chromosome, i.e. 6.1 refers to the first region containing a putative lethal recessive haplotype located along chromosome 6. *P* values are given for the probability of observing no homozygotes within the population given the number expected based on the frequency of the haplotype ($P (O[ H ] = 0 |E[ H ])$), the probability of observing no homozygous offspring given the expected number from matings between a carrier sire and a carrier maternal grand sire ($P\left( {O[ H ] = 0 | S, MGS Carriers} \right)$) and the significance of mating status (MS) using the *S* × *MGS* model. $R$ is the relative reduction in total number born as a result of matings between two carriers compared to matings between a carrier and a non-carrier. The number of standard errors that $R$ was from the expectation of a lethal recessive effect and from no effect are given. ^‡^ indicates a departure from the expectation of a lethal effect. *P* values are nominal, with the haplotypes that achieve Bonferroni corrected significance (*P* < 3.25 × 10^−4^) for MS highlighted in italics. Results arising from the concurrent assessment of two putative lethal recessive haplotypes are denoted by ^†^

**Table 4:** Locations and frequencies of the six putative lethal recessive haplotypes and locations of the regions in which they reside

Region	Chromosome	Region position (Mb)	Haplotype position (Mb)	Haplotype frequency
1.1	SSC1	136.78–137.50	136.78–137.46	0.018
1.2	SSC1	187.04–187.58	187.12–187.53	0.018
6.1	SSC6	24.41–28.28	24.41–24.56	0.020
10.1	SSC10	25.78–26.48	25.91–26.48	0.016
14.1	SSC14	60.26–60.62	60.38–60.62	0.017
14.2	SSC14	116.38–116.86	116.56–116.80	0.015

Two haplotypes on SSC1 (located in regions 1.1 and 1.2) were identified as having a putative lethal recessive effect using the *S* × *MGS* model. The effect of MS for the assessed haplotype within the 1.1 region was statistically significant after applying a Bonferroni correction, although the 1.2 region had an $R$ that was just outside of the allowable threshold (Table 3). Individuals that carried both haplotypes in regions 1.1 and 1.2 had an $R$ (0.122) that was closer to that expected for a lethal recessive effect compared to the single haplotypes. The *r* ^2^ between the haplotypes in regions 1.1 and 1.2 was 0.41; the *r* ^2^ between each of the SNPs within these haplotypes are in Additional file 2: Figure S1. Single putative lethal recessive haplotypes were detected in regions located on SSC6 (6.1) and SSC10 (10.1), with evidence provided from both the *S* × *D* and the *S* × *MGS* models. SSC14 provided evidence for haplotypes in two regions (14.1. and 14.2; *r* ^2^ = 0.41) that had a putative lethal recessive effect after applying a Bonferroni correction. The *r* ^2^ between each of the SNPs within these haplotypes are provided in Additional file 3: Figure S2. Further information regarding the regions that contain putative lethal recessive haplotypes with an effect on TNB is in Additional file 4.

### SNP-by-SNP approach with an effect on TNB

Analysing all individuals and only those individuals genotyped on the MedD chip using a SNP-by-SNP approach failed to detect any alleles with a putative lethal recessive effect on TNB.

### Analysis of individuals genotyped at MedD only for an effect on TNB

A region (13.1) on SSC13 was identified as potentially harbouring a nominally significant putative lethal recessive haplotype (for more information see Additional file 4). This haplotype was further examined using the full dataset with all individuals, which revealed that three imputed LowD individuals were predicted as being homozygous. Two of the three homozygous individuals had their sire and all grandparents genotyped at MedD, with LowD information available for the dam, while the other homozygous individuals had their sire, dam and male grandparents genotyped at MedD. LowD markers adjacent to the region of interest were positioned at 34.48, 36.84 and 44.56 Mb.

### Effect on NBA as a proportion of TNB

No evidence was found for any putative haplotypes or SNPs with a lethal recessive effect on the NBA/TNB using all individuals or only the individuals genotyped at MedD, suggesting that only effects on embryonic mortality were detectable in the current dataset.

## Discussion

We have developed a formal test for the detection of lethal recessive variants in species that produce large full-sib families, such as pigs, birds, fish and trees, with *P* values used to identify a putative lethal recessive haplotype that are valid in a hypothesis-driven scientific context. However, in a commercial setting, a dynamic approach could be adopted, with ongoing monitoring of putative regions as more data are accumulated, and by applying alternative thresholds to obtain the maximum benefit for the population involved. The proposed methodology detected six regions, relating to at least four putative lethal recessive variants. The nucleus population analysed in this study has been selected for maternal performance traits and therefore has been subjected to natural and artificial selection pressure against carriers of lethal recessive variants, which has limited the increase in the frequency of any associated haplotypes, making their detection more difficult. Therefore, the ability to detect the existence of putative lethal recessive variants in this population suggests that such variants are likely to be pervasive across other selectively bred populations. Indeed, results from an earlier much smaller study support this conclusion for pigs [17].

### Procedure adopted for identifying lethal recessive haplotypes

When using haplotype frequency (Step 1A) to determine the expected number of homozygotes, random mating was assumed. We observed more than 1.7 million segregating haplotypes that were not present in the homozygous state in this population, but only 0.08% of these were considered as unusual ($P (O[ H ] = 0 |E[ H ])$ < 0.05) because most of the 1.7 million haplotypes segregated at relatively low frequencies and the probability of not observing a homozygous haplotype is a decreasing function of $q$ (since the expected number of homozygous haplotypes is an increasing function of $q$). Thus, the lack of homozygotes is considered unusual only for haplotypes that segregate at a sufficiently high frequency. This method alone was not sufficient to identify lethal haplotypes since most candidate haplotypes identified in Step 1A produced no evidence of an effect on TNB or NBA/TNB. Thus, the observed lack of homozygous haplotypes may have occurred by chance, due to an artefact of imputation, non-random mating, or selection prior to genotyping.

The putative lethal recessive haplotypes that were classified as candidate haplotypes using the within-family analysis approach of Steps 1B and 1C were also classified as candidates using the population-wide haplotype frequency of Step 1A. This overlap between the three methods used for Step 1 confirms the validity of the adopted approaches for detecting putative lethal recessive effects.

In the population studied, typically only the potential selection candidates were genotyped, providing a bias towards individuals that were genetically superior for traits under artificial selection, and thus the genetically inferior individuals, which may have been homozygous for a particular haplotype, were not genotyped. There was also potentially a disinclination to genotype individuals that were born within litters with a lower TNB or NBA/TNB, as this was a line selected for maternal traits.

The genomic regions that were ultimately detected as containing putative lethal recessive variants, had haplotype frequencies that ranged from 1.5 to 2.0%. These haplotype frequencies were generally lower than those estimated for recessive variants detected in cattle [14, 16]. The centralised control of breeding decisions and the control of the rate of inbreeding within the population studied, may have limited the spread of lethal recessive variants. Assuming $n$ unlinked lethal haplotypes with a common frequency $q$, then the proportion of matings which would be *C* × *C* for the same variant, $m$, can be calculated as:

8 $$m = 1 - (1 - \left( {2pq} \right)^{2} )^{n} ,$$

where $p = 1 {-} q$. Given a haplotype frequency of $q$  = 0.02 for each of the four putative lethal recessive variants detected, then it is expected that 61 matings out of 10,000 will have occurred between carriers for the same putative lethal recessive variant. Based on the mean TNB in this population, it is expected that these four variants potentially cause the loss of 0.15% of all embryos. Thus, identification and management of lethal recessive variants is desirable since a doubling of the haplotype frequencies of these four lethal recessives variants would almost quadruple the number of affected matings, i.e. to 239 out of 10,000, causing the loss of 0.58% of all embryos in the population.

### Putative lethal recessive haplotypes with an effect on TNB

For the two putative lethal recessive haplotypes on SSC1, the number of litters produced from carrier sires and carrier dams (6 and 9, respectively) was small, which limited the power to detect an effect on TNB using the *S* × *D* model. However, when using the *S* × *MGS* model to examine the TNB from litters with carrier sires and dams sired by carrier MGS, the number of *C* × *C* litters (150 and 122, respectively) was much larger. Comparison of the observed *P* values for all putative lethal recessive haplotypes from the *S* × *D* and the *S* × *MGS* models in Tables 2 and 3 suggests that the *S* × *MGS* model has more power to detect lethal recessive effects.

The most interesting results for SSC1 concerned double carriers of both putative lethal haplotypes. The physical distance between the two regions was ≈ 50 Mb, which corresponds to a predicted genetic distance of 10 cM [24] to 24 cM [25]. The double carrier $R$ was much closer to that expected for a single lethal recessive variant compared to either of the single carrier $R$ for regions 1.1 or 1.2 on SSC1. One might have expected two separate variants, each with a negative effect and each located near the region 1.1 or 1.2. However, based on the observed $R$, the linkage disequilibrium between the two haplotypes (*r* ^2^ = 0.41) and the lack of an additional effect above that expected for a single region, it is more likely that there is a single putative lethal recessive variant located in between the two regions. This variant may have not been detectable at its true location, since the short length of haplotypes used may not have fully capture the genetic heterogeneity at that location. An alternative explanation may involve an epistatic lethal effect between the two variants, which may explain why no other putative lethal haplotypes were found between the two regions.

Located in-between the two haplotypes on SSC1, a QTL was reported at approximately 158 Mb that explained roughly 3% of the dominance variance for TNB in Large White pigs [26]. A genome-wide association study identified a QTL for TNB at 173.58–173.66 Mb and a QTL for NBA at 182.39–182.51 Mb using a Yorkshire, Duroc and Landrace composite breed [27]. Both these QTL are within 10 Mb of region 1.2 on SSC1. The use of next-generation sequencing, focused primarily between the two regions of interest in carrier individuals, may help identification of the causal variant(s) and may provide an attribution of biological causality.

The physical distance between the two regions identified on SSC14, 14.1 and 14.2, was approximately 56 Mb which corresponds to a genetic distance of 16 [24] to 30 cM [25]. Using the *S* × *D* model, the double carrier $R$ was closer to the expectation for a lethal recessive variant than that for single carriers. However, examination of the pairwise linkage disequilibrium *r* ^2^ values (see Additional file 3) suggested a potential misalignment of the SNP positioned at 111,786,069 Mb within region 14.2, since we found that it had *r* ^2^ ranging from 0.21 to 0.43 with three of the five SNPs located in region 14.1. Such a misalignment may result in the detection of an additional signal if the misaligned SNP was in linkage disequilibrium with the causal lethal recessive variant. Previously, QTL for TNB that overlap with region 14.1 were identified in Large White pigs [3].

The $R$ calculated for all putative lethal recessive haplotypes were lower than those expected for a lethal recessive variant. There are a number of potential causes for the observed $R$ not reaching the expected ratio. First, the lethal recessive variant may not be in linkage equilibrium with the detected haplotype. In this case, recombination between the variant and the haplotype may reduce the effect of the haplotype on TNB, with some carriers of the haplotype not carrying the lethal recessive variant and thus their litter size being similar to that of non-carriers. Second, when applying the *S* × *MGS* model, selection may have occurred against dams that were carriers since they would have had a reduced TNB. This would reduce the haplotype frequency in dams below that assumed in the model, thereby reducing the observed $R$.

### SNP-by-SNP approach and the use of individuals genotyped at MedD only

Analysing the data using a SNP-by-SNP approach failed to detect any lethal recessive alleles. Human populations have a large diversity of diplotypes (haplotypes pairs) [28] compared to artificially selected livestock population. As a result, a SNP-by-SNP approach ultimately fails to capture all the underlying variation, resulting in type II errors. SNPs with a low MAF are typically under-represented on the PorcineSNP60 BeadChip [29], which reduces the ability of genotype-based data to tag rarer variants, such as those with a lethal recessive effect. This hypothesis was supported by the results obtained in our study.

Analysis of individuals genotyped at MedD only failed to detect any of the lethal recessive regions that were identified using the all individuals, which was potentially due to an insufficient number of carrier individuals or offspring produced from matings between carriers to qualify as candidate haplotypes. The inclusion of imputed LowD individuals was therefore vital to maximising the power provided by the genomic data to detect lethal recessive effects, as well as negating false positives, which may have arisen otherwise.

The haplotype identified in region 13.1 on SSC13 provided background evidence of a negative effect on TNB, although it was unlikely to be a lethal recessive effect, based on the additional validation conducted using the imputed LowD individuals. Evidence to support the accuracy of the imputed data in this region was provided by both the availability of close ancestors with MedD information and the proximity of LowD markers to the region of interest. A 27-Mb region that overlaps with region 13.1 is known to affect TNB and NBA [30], which was attributed to inbreeding depression based on runs of homozygosity near a QTL [31]. In our study, region 13.1 was identified as containing candidate haplotypes based on lack of homozygosity and could therefore be due to selection. This region may contain multiple variants that influence litter size. As a result, different populations and breeds may harbour different variants, some with a negative additive effect and others with a lethal recessive effect.

### The benefits of using haplotypes with a varying number of SNPs

Among the six putative lethal recessive haplotype regions detected, three were detected using a 3-SNP length, all were detected using a 5-SNP length and five were detected using a 10-SNP length. Although 5-SNP lethal recessive haplotypes were identified in each of the six regions, this could not have been predicted prior to the study. The length of the haplotype that is required to detect a lethal recessive effect will depend on the extent of linkage disequilibrium between the variant and the haplotype, the actual and effective size of the population, the age of the variant, the SNP density, the proximity of SNPs to the true lethal recessive variant and the recombination rate in the region of interest. Therefore, we recommend the use of haplotypes with varying lengths to detect lethal recessive effects in future studies.

### Putative haplotypes with an effect on NBA/TNB

We found no haplotypes that had an effect on NBA as a proportion of TNB. However, this does not mean that there are no recessive haplotypes that cause foetal death in this population. The frequency of such lethal recessive haplotypes could be too low to detect an effect on foetal mortality in a population of the size used in this study. Offspring that are born dead could potentially be assessed using a homozygosity mapping approach [13, 32].

## Conclusions

This study demonstrates the efficacy of using haplotypes to identify regions of the porcine genome that carry a putative lethal recessive effect on embryonic mortality. Evidence of such an effect was found for four porcine chromosomes (SSC1, 6, 10 and 14). These results were obtained by using 5660 individuals genotyped at 60 K and a further 12,534 individuals genotyped at low density (402 SNPs). With continuing declines in the cost of genotyping, this approach to manage genetic diversity will be practical, measurable and increasingly affordable. We show that the use of haplotypes is more powerful to detect lethal recessive effects than a SNP-by-SNP approach. The exact locations and underlying biological processes that cause the lethal effects identified in this study require additional research and the use of next-generation sequencing. However, the methodology that we used in this study allows the direct identification of individuals that are carriers of a putative lethal recessive haplotype. Such carriers can then be removed from selection programs or mated to non-carrier individuals only, thereby improving overall reproductive performance.

## Additional files

**Additional file 1.** Derivation of Eq. (4) applied during Step 1C to determine the probability of observing no homozygous offspring given the expected number of homozygous individuals from matings between a carrier sire and a carrier maternal grand sire.

**Additional file 2: Figure S1.** Heatmap of the linkage disequilibrium *r* ^2^ values between each of the SNPs located within regions 1.1. and 1.2 on SSC1.

**Additional file 3: Figure S2.** Heatmap of the linkage disequilibrium *r* ^2^ values between each of the SNPs located within regions 14.1. and 14.2 on SSC14.

**Additional file 4.** Detailed results on the putative lethal recessive haplotypes associated with TNB.
