# Supplementary material for: Use of haplotypes to identify regions harbouring lethal recessive variants in pigs
Source: Genet Sel Evol. 2017 Jul 14;49:57. doi: 10.1186/s12711-017-0332-3 (PMC5512953; doi:10.1186/s12711-017-0332-3)
Supplement: Supplementary file 1 — Additional file 1. Derivation of Eq. (4) applied during Step 1C to determine the probability of observing no homozygous offspring given the expected number of homozygous individuals from matings between a carrier sire and a carrier maternal grand sire. [file 12711_2017_332_MOESM1_ESM.docx]

**Additional file 1. The derivation of equation (4) applied during step 1C to determine the probability of observing no homozygous offspring given the expected number from matings between a carrier sire and a carrier maternal grand sire.**

Assume the null hypothesis of non-lethality of the haplotype of interest (say ‘AA’), which has a frequency of *q* in the population. The dam offspring of the carrier maternal grand sire (MGS) has probabilities ½*q*, ½ and ½(1-*q*) of being homozygote for ‘AA’, a carrier heterozygote and a non-carrier. If the dam is an ‘AA’ homozygote, the offspring of a mating with a carrier sire has probability ½ of not being a homozygote ‘AA’, and with *n_m_* offspring the probability of not observing an ‘AA’ homozygote is ½*^nm^*. If the dam is a carrier heterozygote the probability of not observing an ‘AA’ homozygote is ¾*^nm^*, and if the dam is not a carrier the probability is 1. Therefore the probability given a mating between a carrier sire and the offspring of a carrier MGS is ½(*q*½*^nm^* + ¾*^nm^* + (1-*q*)). Making the assumption that the different mating pairs can be approximated as being independent the probability is:

$$\prod_{m=1}^{M} \left( \frac{{0.5}^{n_{m}}q+{0.75}^{n_{m}}+(1-q)}{2} \right)$$

where there was a total of $M$ litters and litter $m$ has $n_{m}$ haplotyped offspring
