# Supplementary figures and images for: Use of haplotypes to identify regions harbouring lethal recessive variants in pigs
Source: Genet Sel Evol. 2017 Jul 14;49:57. doi: 10.1186/s12711-017-0332-3 (PMC5512953; doi:10.1186/s12711-017-0332-3)

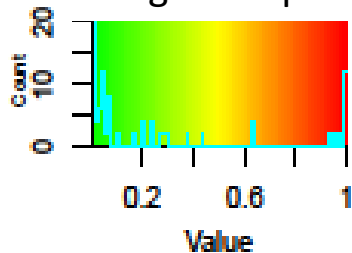

Mb Position

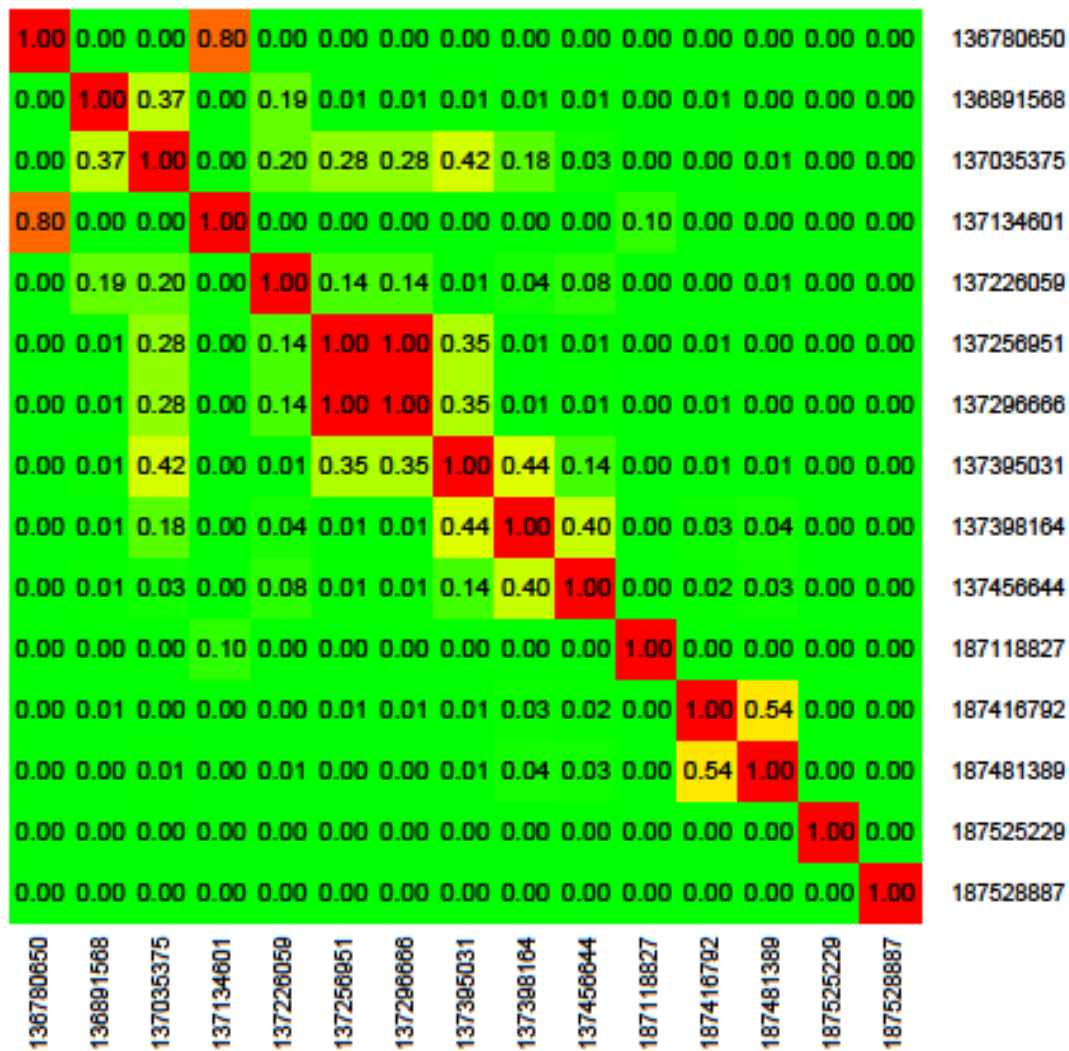

Haplotype in  
Region 1.1

Haplotype in  
Region 1.2

Supplement: Supplementary file 2 — Additional file 2: Figure S1. Heatmap of the linkage disequilibrium r 2 values between each of the SNPs located within regions 1.1. and 1.2 on SSC1. [file 12711_2017_332_MOESM2_ESM.pdf]

Linkage disequilibrium  $r^2$

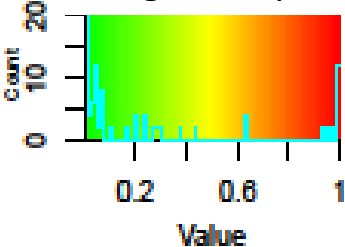

Mb Position

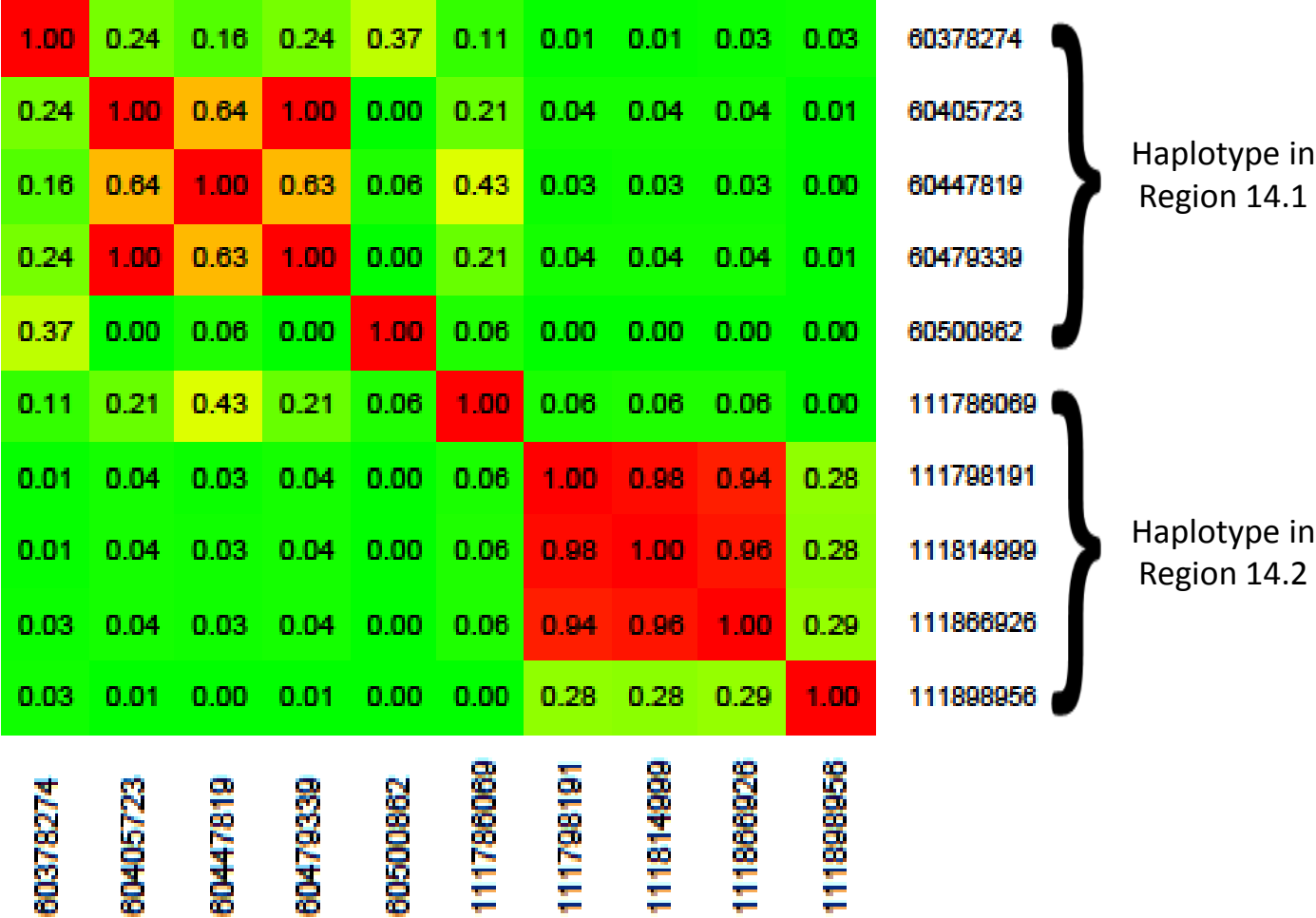

Supplement: Supplementary file 3 — Additional file 3: Figure S2. Heatmap of the linkage disequilibrium r 2 values between each of the SNPs located within regions 14.1. and 14.2 on SSC14. [file 12711_2017_332_MOESM3_ESM.pdf]
