# Supplementary material for: Use of haplotypes to identify regions harbouring lethal recessive variants in pigs
Source: Genet Sel Evol. 2017 Jul 14;49:57. doi: 10.1186/s12711-017-0332-3 (PMC5512953; doi:10.1186/s12711-017-0332-3)
Supplement: Supplementary file 4 — Additional file 4. Detailed results on the putative lethal recessive haplotypes associated with TNB. [file 12711_2017_332_MOESM4_ESM.docx]

**Additional file 4. Detailed results for putative lethal recessive haplotypes associated with TNB**

**Detailed results for putative lethal recessive haplotypes associated with TNB using all individuals**

*Chromosome 1*

A 0.72 Mb region (region 1.1) on SSC1, located between 136.78 – 137.50 Mb, was identified as containing candidate haplotypes of 5-SNP and 10-SNP lengths. There were 702 heterozygous carriers of a 10-SNP length candidate haplotype located between 136.78 and 137.46 Mb. This haplotype had a frequency of 0.018 which provided an expectation of 6.14 homozygous individuals. This expectation provided a significant $P\left( O[H]=0 \right|E[H])$ value of 2.15 x 10^-3^. Both $P\left( O\left[ H \right]=0 | S, D Carriers \right)$ and $P\left( O\left[ H \right]=0 | S, MGS Carriers \right)$ were not significant (*P* ≥ 0.05), with 7 and 8 haplotyped offspring produced respectively. Only 6 *C* x *C* litters were produced from sire and dam matings, providing low power for detection using the *S* x *D* model. There were 150 litters yielded from carrier sires and carrier MGS for comparison with 8,662 *C* x *NC* litters. The fitting of the *S* x *MGS* model resulted in a *P*-value for MS of 6.99 x 10^-5^, which remained significant after applying a Bonferroni correction. The predicted values for the TNB of *C* x *C* matings was 10.15 and for the *C* x *NC* matings was 11.19, providing a relative reduction ($R$) of 0.092. The standard error of $R$ was 0.023, placing $R$ 1.39 standard errors away from that expected for a lethal recessive effect involving a carrier sire and carrier MGS.

Approximately 50 Mb further along SSC1, a second region (1.2) containing a putative lethal recessive haplotype was identified. This region contained both 3-SNP and 5-SNP candidate haplotypes and was located between 187.04 and 187.58 Mb. A 5-SNP haplotype positioned at 187.12 – 187.53 Mb had a frequency of 0.018 which provided a statistically significant $P\left( O[H]=0 \right|E[H])$ value of 1.17 x 10^-3^. There were 9 sire and dam *C* x *C* litters produced, with MS found not to be significance using the *S* x *D* model. A total of 122 litters were produced from carrier sires and carrier MGS and when applying the *S* x *MGS* model, MS was statistically significant with a *P*-value of 7.11 x 10^-3^. The TNB predicted value for *C* x *C* matings was 10.66 and for *C* x *NC* matings was 11.46. These predicted values equated to an $R$ of 0.070 with a standard error of 0.026. The observed effect was 2.13 standard errors away from the expectation of 0.125, which was outside of that expected for a lethal recessive haplotype.

Further analysis was made of those individuals which were carriers of both haplotypes within the two regions, 1.1 and 1.2. There were 513 individuals that were carriers for both the haplotype at 136.78 – 137.50 Mb and the haplotype at 187.12 – 187.53 Mb. Based on the observed haplotype frequencies it was calculated that the $r^{2}$ was 0.409. A total of 70 litters were produced from matings between double carrier sires and double carrier MGS. After fitting the *S* x *MGS* model, MS had a *P*-value of 2.07 x 10^-4^ which was statistically significant after applying a Bonferroni correction. The predicted TNB for *C* x *C* matings was 10.45 and for the *C* x *NC* matings was 11.90. The $R$ for the *S* x *MGS* model was 0.122 with a standard error of 0.033. This placed $R$ within 0.09 standard errors of that expected of a lethal recessive effect.

*Chromosome 6*

A relatively wide 3.87 Mb region (6.1) containing 3-SNP, 5-SNP and 10-SNP candidate haplotypes was identified on SSC6, located between 24.41 – 28.28 Mb. A 10-SNP haplotype located between 24.41 – 24.56 Mb with a frequency of 0.020, generated a statistically significant $P\left( O[H]=0 \right|E[H])$ value of 7.84 x 10^-4^. Sixteen haplotyped offspring were produced from matings between carrier sires and carriers dams giving a statistically significant $P\left( O\left[ H \right]=0 | S, D Carriers \right)$ value of 0.01. Eight litters were produced from matings between carrier sires and carrier dams for comparison with 635 *C* x *NC* matings. The *S* x *D* model produced an indicative significance for MS of 0.056, with a predicted TNB for *C* x *C* matings of 10.51 and 12.46 for *C* x *NC* matings. $R$ was 0.157 with a standard error of 0.082, which was 1.13 standard errors away from the expected lethal effect. There were 72 *C* x *C* litters produced from carrier sires and carrier MGS with 4,831 *C* x *NC* matings available for comparison. Applying the *S* x *MGS* model resulted in a significant *P*-value for MS of 3.25 x 10^-3^, with predicted values of 9.98 for the TNB from *C* x *C* and 11.13 for *C* x *NC*. $R$ was 0.103 with a standard error of 0.035, revealing that the observed effect was 0.62 standard errors away from that expected.

*Chromosome 10*

A region (10.1) spanning 25.78 – 26.48 Mb was identified as containing both 5-SNP and 10-SNP candidate haplotypes on SSC10. A 5-SNP haplotype between 25.91 – 26.48 Mb had a frequency of 0.016 and generated a significant $P\left( O[H]=0 \right|E[H])$ value of 5.30 x 10^-3^. There were 22 haplotyped offspring from matings between carrier sires and carrier dams which equated to a significant $P\left( O\left[ H \right]=0 | S, D Carriers \right)$ value of 1.78 x 10^-3^. The $P\left( O\left[ H \right]=0 | S, MGS Carriers \right)$ value was significant (*P* = 4.74 x 10^-6^) with 97 haplotyped offspring produced from carrier sires and carrier MGS. There were 13 litters produced from carrier sires and carrier dams, with a significant effect observed for MS with a *P*-value of 0.048. The predicted values for the TNB were 9.75 for *C* x *C* matings and 11.56 for *C* x *NC* matings. $R$ was 0.157 with a standard error of 0.079, placing the effect 1.18 standard errors away from the expectation of a lethal recessive effect. Carrier sires and carrier MGS produced 88 *C* x *C* litters for comparison with 4,841 *C* x *NC* litters. The *S* x *MGS* model revealed a significant *P*-value for MS of 7.69 x 10^-3^, with predicted TNB values for *C* x *C* matings of 10.61 and 11.56 for *C* x *NC* matings. $R$ was 0.083 with a standard error of 0.031, placing $R$ 1.37 standard errors away from the expectation of a lethal recessive effect.

*Chromosome 14*

Two regions containing putative lethal recessive haplotypes were identified on SSC14. The first was a relatively short 0.36 Mb region (14.1) spanning 60.26 – 60.62 Mb, containing both 5-SNP and 10-SNP candidate haplotypes. A 5-SNP candidate haplotype covering 60.38 – 60.62 Mb was found to have had a frequency of 0.017 which equated to a significant $P\left( O[H]=0 \right|E[H])$ value of 1.77 x 10^-3^. There were 39 haplotyped offspring from matings between carrier sires and carrier dams, producing a significant $P\left( O\left[ H \right]=0 | S, D Carriers \right)$ value of 1.34 x 10^-5^. $P\left( O\left[ H \right]=0 | S, MGS Carriers \right)$ was found to be significant with 48 haplotyped offspring generating a *P*-value of 8.67 x 10^-3^. A total of 32 *C* x *C* litters were produced from carrier sires and carrier dams for comparison with 678 *C* x *NC* matings, with MS found to be statistically significant (*P* = 2.03 x 10^-5^). After applying a Bonferroni correction MS remained statistically significant. The predicted values for the TNB were 9.83 for *C* x *C* matings and 12.14 for *C* x *NC* matings and $R$ was calculated as 0.190 with a standard error of 0.045. This places the observed effect 1.34 standard errors away from that expected of a lethal recessive effect. Where the matings involved a sire and a MGS there were a total of 322 *C* x *C* litters and 7,497 *C* x *NC* litters. Comparing the TNB of *C* x *C* matings with *C* x *NC* matings produced a significant *P*-value for MS of 6.28 x 10^-10^, which remained statistically significant after applying a Bonferroni correction. The predicted TNB for *C* x *C* matings was 10.44 with the *C* x *NC* matings having a predicted TNB of 11.61. $R$ was 0.101 with a standard error of 0.017, which places the observed effect 1.45 standard errors away from that expected of a lethal recessive effect.

A second region (14.2) containing a putative lethal haplotype was also identified on SSC14, located between 116.38 Mb - 116.86 Mb. Candidate haplotypes of 3-SNP, 5-SNP and 10-SNP length were all found within this region, with a 5-SNP haplotype examined at 116.56 – 116.80 Mb. This haplotype had a frequency of 0.015 which produced a statistically significant $P\left( [O]=0 \right|E[H])$ value of 0.015. The number of haplotyped offspring produced from carriers were significant with $P\left( O\left[ H \right]=0 | S, D Carriers \right)$ = 5.64 x 10^-3^ and $P\left( O\left[ H \right]=0 | S, MGS Carriers \right)$ = 0.019, with 18 and 42 offspring produced respectively. There were 19 litters produced from matings between carrier sires and carrier dams. Applying the *S* x *D* model gave a significant *P*-value for MS of 9.71 x 10^-4^. The predicted TNB for *C* x *C* matings was 9.88 and was 12.23 for *C* x *NC* matings. $R$ was calculated as 0.196 with a standard error of 0.059. This placed $R$ 0.91 standard errors away from that expected of a lethal recessive effect. There were 213 *C* x *C* and 4,557 *C* x *NC* litters available for analysis from carrier sires and carriers MGS. The *S* x *MGS* model produced predicted TNB values of 10.89 for *C* x *C* and 11.89 for *C* x *NC*, with MS found to be statistically significant with a *P*-value of 1.01 x 10^-5^. After applying a Bonferroni correction, MS remained significant. $R$ was 0.084 with a standard error of 0.019, which was 2.11 standard errors away from that expected of a lethal recessive effect. This placed the observed effect just outside the predefined threshold of 1.96 standard errors for the classification of a putative lethal recessive when considering the *S* x *MGS* model.

Individuals which were carriers of both putative lethal recessive haplotypes in region 14.1 and region 14.2 were considered. A total of 376 individuals were identified as being carriers of both of these haplotypes, with an $r^{2}$ value calculated as 0.405 between the two haplotypes. There were 17 litters produced by double carrier sire and double carrier dam matings. The *S* x *D* model produced a statistically significant *P*-value for MS (1.02 x 10^-4^). After applying a Bonferroni correction, MS remained statistically significant. A predicted TNB of 9.41 was calculated for the *C* x *C* matings and for the *C* x *NC* matings the TNB was 12.39. $R$ was 0.240 with a standard error of 0.061. This placed the observed effect 0.16 standard errors away from that expected of a single lethal recessive effect. On applying the *S* x *MGS* model there were 178 *C* x *C* matings and MS was found to be statistically significant (2.97 x 10^-6^). MS remained statistically significant after applying a Bonferroni correction. The TNB predicted values were 10.78 for *C* x *C* matings and 11.94 for *C* x *NC* matings. $R$ was calculated as 0.097, with a standard error of 0.021, placing the effect 1.33 standard errors away from the expected lethal recessive effect.

**Detailed results for putative lethal recessive haplotypes associated with TNB using only MedD individuals**

Chromosome 13

A 5-SNP haplotype was identified between 37.12 – 37.31 Mb and produced a significant $P\left( OH=0 \right|E[H])$ value of 8.76 x 10^-3^. The *S* x *D* model had a significant *P*-value for MS of 1.01 x 10^-3^. $R$ was 0.193 with a standard error of 0.058, placing $R$ 0.97 standard errors away from that expected of a lethal haplotype. MS was not statistically significant (*P* ≥ 0.05) using the *S* x *MGS* model.
